# Synergetic Effect of Silver Nanoparticles and *UVC* Irradiation on *H2AX* Gene Expression in TK6 Cells 

**DOI:** 10.22074/cellj.2019.5898

**Published:** 2019-02-25

**Authors:** Tahereh Zare, Reza Fardid, Samaneh Naderi

**Affiliations:** 1 Department of Radiology, School of Paramedical Sciences, Shiraz University of Medical Sciences, Shiraz, Iran; 2 Ionizing and Non-Ionizing Radiation Protection Research Center (INIRPRC), Shiraz University of Medical Sciences, Shiraz, Iran; 3 Diagnostic Laboratory Sciences and Technology Research Center, School of Paramedical Sciences, Shiraz University of Medical Sciences, Shiraz, Iran

**Keywords:** Genotoxicity, *H2AX*, Nanoparticles, Silver, Ultraviolet

## Abstract

**Objective:**

The use of nanoscale particles, for instance silver nanoparticles (Ag NPs) has considerably increased recently. Since Ag NPs can be transmuted into silver ions; the toxicity and genotoxicity of these NPs along with other external factors such as ultraviolet type C (UVC) irradiation must be evaluated. In the present study, the aim was to investigate the genotoxic effects Ag NPs and UVC co-exposure on human lymphoblastoid TK6 cells.

**Materials and Methods:**

In this experimental study, Ag NPs (~20 nm) were purchased from US Research Nanomaterials Inc. and *H2AX* gene expression was evaluated using quantitative real time polymerase chain reaction (qRT-PCR), 1 and 24 hours post Ag NPs and UVC treatment.

**Results:**

Results showed that treatment of TK6 cells with different Ag NP concentrations without exposure to UVC can reduce *H2AX* gene expression, but treatment of these cells with Ag NPs in combination UVC irradiation can reduce viability that leads to a synergistic increase in the amount of *H2AX* gene expression.

**Conclusion:**

According to our findings, Ag NPs can act to sensitize cells to UVC radiation when used for cancer treatment. So, combination of Ag NPs and UVC irradiation could be used in radiotherapy.

## Introduction

In recent years nanotechnology has attracted a great deal of attention in numerous fields such as biochemistry, physics, biology, material science etc. (1). Over past decades, silver (Ag) has been the subject of extensive research for antibacterial and anti-fungal purposes (2). Ag is of particular interest in health care, the food industry, water purification, and household products (3, 4). One of the applications of Ag NPs in medicine is for cancer treatment (5, 6). Nowadays the use of Ag at the nano scale has increased due to recent development in nanotechnology. Previously, silver was considered as a nontoxic metal; however, recent studies have shown that Ag is the second most harmful metal after mercury to freshwater fish and invertebrates (7-9). Nanoparticles (NPs) are commonly considered to be more toxic than micro-sized particles due to their individual physicochemical characteristics, and the small size of the particles (10). Therefore, the toxicity of Ag nanoparticles (Ag NPs) must be determined for safe and effective usage, especially in mammalian cells because Ag NPs dissolve into Ag ions (11), and can directly bind to RNA polymerase, leading to the inhabitation of RNA polymerase activity, and over all RNA transcription. This is process is separate from the cytotoxic effects of Ag ions (12). 

Sunlight ultraviolet (UV) radiation can have harmful effects on all living organisms including animals and humans (13). Generally, UV radiation is divided in to three segments based on the wavelength: ultraviolet type A (UVA) (320-400 nm), ultraviolet type B (UVB) (280-320 nm) and ultraviolet type C (UVC) (200-280 nm). UVA and UVB penetrate the ozone layer and have significance physiological effects (14), but UVC is absorbed by the ozone layer and cannot reach the surface of the earth. One of the main applications of UVC is in disinfection technologies for water and liquid food products due to its advantages over alternatives (15). UVC has an antimicrobial effect on different types of microorganisms due to photochemical changes induced in the pyrimidines of DNA and RNA (16). DNA breaks produced by UV radiation, prevent DNA replication and transcription leading to impaired cellular function, and eventually cell death (17). It can therefore also be used in treating cancer. The lethal effects of UV radiation depend on the radiation doseage, and the capability of the cell to repair the damage (16). 

Many studies have been conducted to investigate the genotoxic effects of Ag NPs by evaluating .-*H2AX* as a marker for detecting DNA double strand breaks (DSB) in mammalian cells (10, 18, 19). However, the genotoxic effects of Ag NPs in combination with UV radiation in humans have not been determined yet.

As mentioned above UVC irradiation and Ag NPs can be used in cancer therapy. In this study we evaluate the effect of Ag NPs as a sensitizer to UVC irradiation in order to kill cancer cells. The present study aimed to investigate the genotoxic effects of Ag NPs in combination with UVC irradiation via evaluating *H2AX* gene expression. To do this, human lymphoblastoid TK6 cells were pretreated with Ag NPs (~20 nm) followed by exposure to UVC irradiation. Next, we measured the *H2AX* gene expression in TK6 cells via quantitative real time polymerase chain reaction (qRT-PCR) to determine the synergistic effects of treatment with Ag NPs plus UVC radiation at 1 and 24 hours post UVC irradiation. 

## Materials and Methods

### Cell culture

In this experimental study, the human lymphoblastoid TK6 cell line was purchased from American Type Culture Collection (ATCC^®^ CRL-8015^TM^) and were maintained in RPMI-1640 medium (Gibco, USA) supplemented with 10% heat incubated fetal bovine serum (FBS, Gibco, USA) and 100 U/ml of penicillin-streptomycin (Gibco, USA), and incubated at 37°C in a humidified atmosphere containing 5% CO_2_. Cells in the exponential growth phase were used in this study. To maintain a culture density of less than 1.2×10^6^ cell/ml, TK6 cells were sub cultured every 2-3 days.

### Ag NPs preparation

Ag NPs (~20 nm: according to Transmission Electron Microscopy (TEM) and XRD pattern by US Research Nanomaterials) were purchased from the US Research Nanomaterials Inc. (Stock#: US1038). To do this study, Ag NPs were suspended in deionized water, and various concentrations were prepared (0, 5, 10 and 15 µg/ml in each well). Ag NPs were immediately sonicating (Hielscher ultrasound technology, UP100H, Germany) before being applied to cells. 

### Treatment with Ag NPs and UVC irradiation

Cells were treated with different concentrations of Ag NPs for 1hr, and then exposed 20 minutes to a germicidal UVC lamp (λ~254 nm) at 1 mW/cm^2^, which was determined with a radiometer (UV-254, Lutron, Taiwan). The cells were returned to an incubator for 1 and 24 hours at 37°C in an atmosphere of 5% CO_2_ in a humidified environment. Non-irradiated cells were handled similar to the UVC irradiated samples, only without being exposed to UVC lamp. 

### Cell viability and MTT assay 

The cells were removed from the incubator twice (1 and 24 hours) post UVC irradiation. Next, the cells were mixed with try-pan blue solution [0.3 % (v/v); 1:1], and cell viability (%) was calculated for all conditions using the following equation: 

Cell viability (%)=(viable cells)/(total cells)×100

The cytotoxicity of Ag NPs in different concentration, and UVC irradiation were investigated by a MTT cell proliferation assay. MTT is reduced to purple formazan crystals in functional mitochondria. The total formazan produced is proportional to the number of viable cells. To perform the MTT assay, TK6 cells at a density of 2.5×10^4^ cells per well were cultured in 96-well culture plates. Then the plate was incubated at 37°C in 5% CO_2_ for 24 hours. The TK6 cells were then treated with different Ag NPs concentration (0, 5, 10 and 15 µg/ml in each well), and irradiated with a germicidal UVC lamp (λ254~ nm). The plate was then returned to the incubator for 1 hour (37°C, 5% CO_2_) after which the MTT solution (5 mg/ ml, Sigma Aldrich, M2128, USA) was added (20 µg in each well) and cells were incubated for 4hrs at 37°C in 5% CO_2_. Following this, the plate was centrifuged (2500 rpm for 40 minutes) and the cell culture medium was discarded. Then dimethyl sulfoxide (DMSO) was added to dissolve the formazan crystals, and the plate was put on a shaker for 30 minutes in a dark room. The absorbance of each well was measured at 545 nm using an ELISA reader (Fax Reader, England). Each experiment was repeated at least three times independently and 0 µg/ml of Ag NPs and UVC-was considered as the control group.

### RNA isolation and quantitative real time polymerase chain reaction

At the 1 hour and 24 hours after UVC irradiation time points, total RNA from each sample was extracted using a RNX-Plus solution (CinaClon Co., Iran) according to the instruction provided by the manufacturer. A Nano drop spectrometer (Helma, USA) was used to determine the quality and concentration of the RNA samples. Approximately 1 µg of total RNA was used for complementary DNA (cDNA) synthesize using RevertAid First Strand cDNA synthesis kit (Thermo Scientific, Lithuania), with a gradient thermal cycler (ASTEC, Japan). cDNA samples were stored at -20°C. The generated cDNA samples were mixed with a master mix (SYBR Green Method with low ROX, Amplicon,) to prepare the qRT-PCR reaction. The qRT-PCR Mixture consisted of 10 µl SYBR green PCR master mix, 0.5 µl forward primer, 0.5 µl reverse primer (10 µ.), and 8 µl nuclease free water. Then 1 µl of the cDNA samples were added to qRT-PCR master mix. The specific primers and reaction conditions used in this study are shown in Table 1. The 48-well plates containing all reagents were briefly centrifuged and analyzed on an ABI Step One Real-Time PCR System (Applied Biosystems, ABI, USA). ß-actin was considered as the housekeeping gene for analyses of this study. 

### Statistical analysis

All experiments were repeated in triplicates. Data are expressed as the mean ± SD. Statistical comparison was done using one-way ANOVA and P<0.05 was considered to be statistically significant. In the cases where the means were compared from the two independent groups, independent t test was used and in the groups that were dependent, paired t test was used.

**Table 1:** Quantitative real-time polymerase chain reaction primers, andreaction conditions for quantitative real time polymerase chain reaction(qRT-PCR)


Gene	Primer sequence (5´-3´)	

H2AFX	F: CAACAAGAAGACGCGAATCA	
	R: CGGGCCCTCTTAGTACTCCT	
ß-actin	F: ATC GTG CGT GAC ATT AAG GAG	
	R: GAA GGAAGG CTG GAA GAG TG	
Three-step qRT-PCR program		
Cycles	Cycles duration	Temperature (°C)
1	2 minutes	95
40	30 seconds	95
	40 seconds	60
	30 seconds	72
1	5 minutes	72


## Results

### Cytotoxicity in combined treatment of TK6 cells with Ag NPs and UVC irradiation

**Fig.1:**
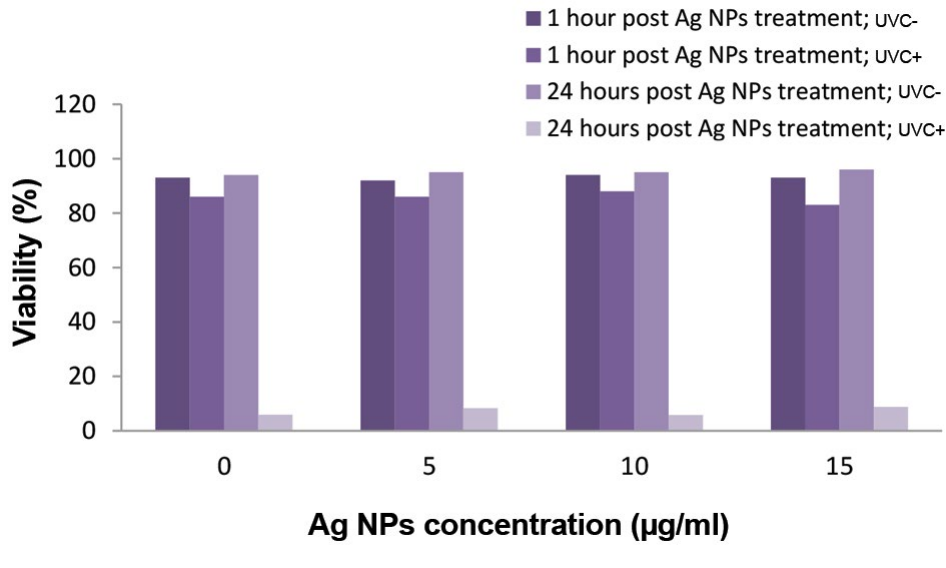
TK6 cell viability after combined treatment with Ag NPs and ultraviolet type C (UVC) irradiation using the try-pan blue assay; cells were harvested 1 hour and 24 hours post UVC exposure (1 mW/cm^2^).

In this study the cytotoxic effect of simultaneous exposure of TK6 cells to Ag and UVC irradiation was examined using try-pan blue dye. In two separate time points following UV irradiation (1 hour and 24 hours) cell viability was reduced at all Ag NP concentrations, which revealed a significant increase in cytotoxicity of Ag NPs with UVC irradiation (Fig.1). 

**Fig.2:**
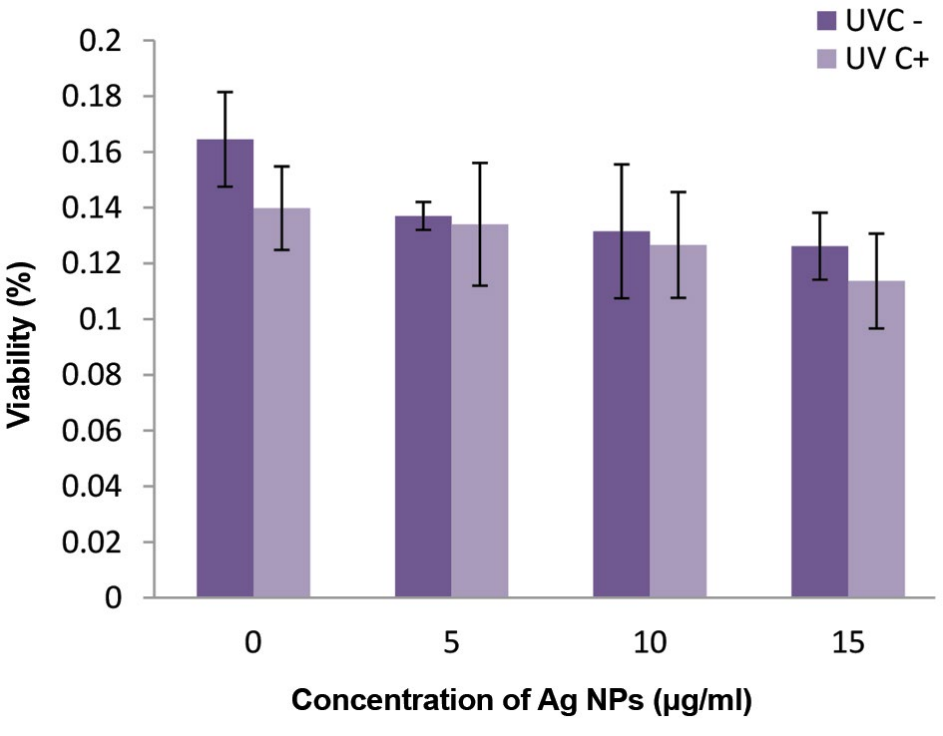
Cytotoxicity of co-exposure to different concentration of Ag NPs and ultraviolet type C (UVC) irradiation (1 mW/cm^2^) by means of MTT assay. 0 µg/ml of Ag NPs and UVC-were considered as the control groups. Data are presented as the mean ± SD.

A MTT colorimetric assay for TK6 cells in the presence of Ag NPs and UVC irradiation was performed. MTT results showed a dose dependent cytotoxicity of Ag NPs with UVC irradiation. On the other hand, various Ag NPs concentrations also showed a significant decrease in cell viability. Also results show, cell viability was reduced by increasing the concentration of the NPs alongside UVC irradiation (Fig.2). These differences were significant in comparison with when each factor was applied separately. Therefore, the MTT test shows increased cytotoxic effects of simultaneous exposure to Ag NPs and UVC irradiation. 

### Genotoxic effects of Ag NPs and UVC irradiation co- treatment on TK6 cells

**Fig.3:**
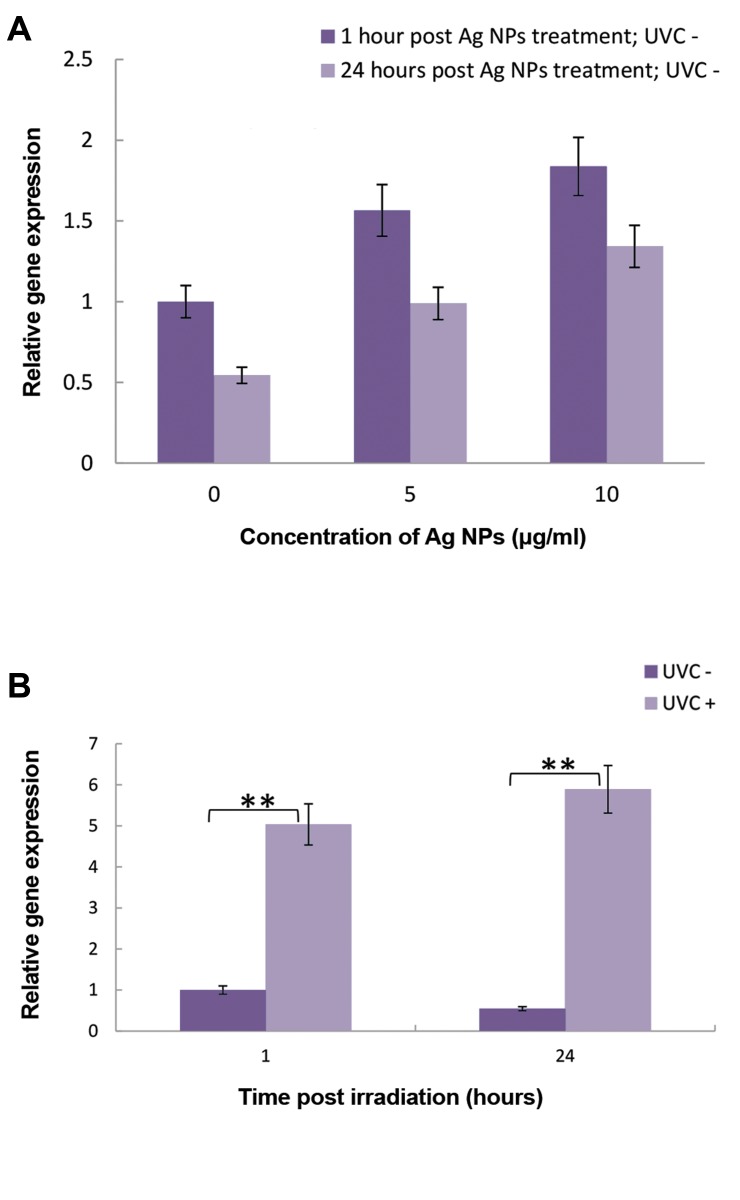
The effect of Ag NPs and ultraviolet type C (UVC) irradiation on *H2AX* gene expression on TK6 cells. *H2AX* gene expression 1 hour and 24 hours after treatment with A. Ag NPs in different concentration (5, 10 and 15 µg/ml) and B. *H2AX* gene expression post UVC irradiation (1 mW/cm^2^) (**; P<0.001). Data are presented as the mean ± SD. P<0.05 were considered as significant.

**Fig.4:**
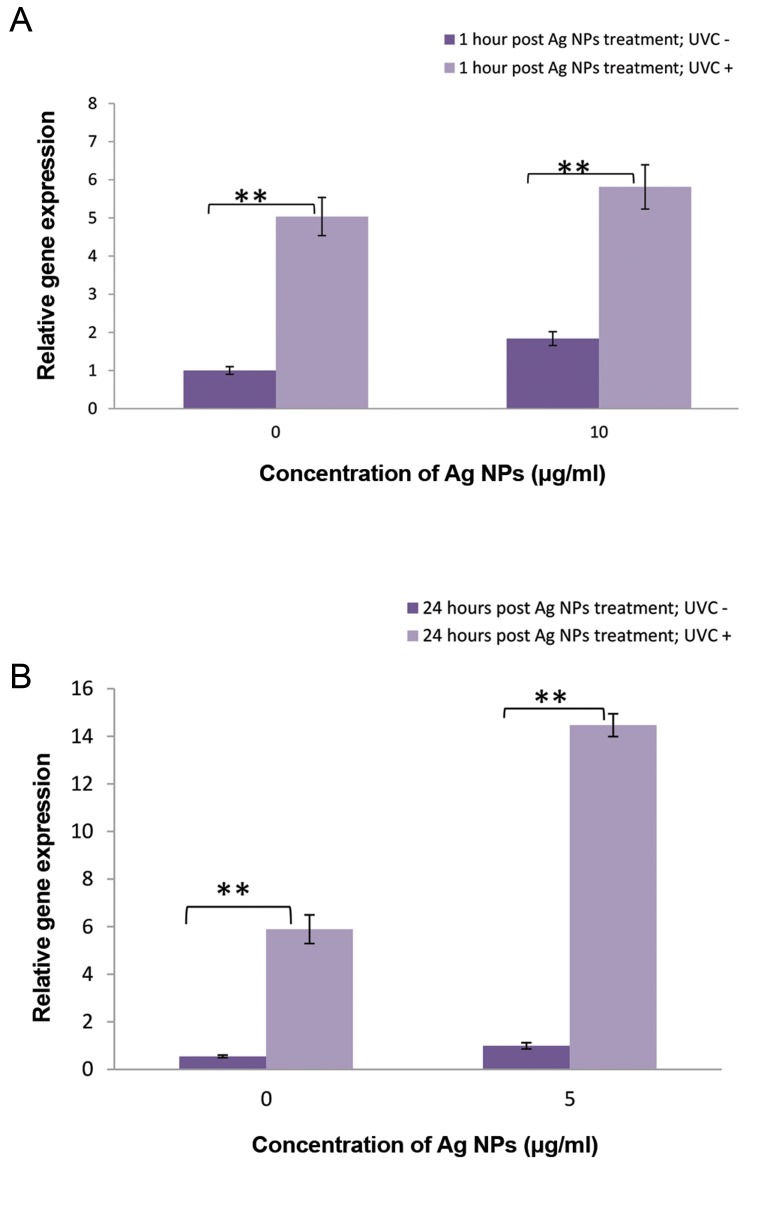
*H2AX* gene expression of Ag NPs and ultraviolet type C (UVC) co- treated cells. TK6 cells were harvested A. 1 hour with 10 µg/ml Ag NPs, and B. 24 hours with 5 µg/ml Ag NPs post exposure to UVC (1 mW/ cm^2^) (**; P<0.001). Data are presented as the mean ± SD. P<0.05 were considered as significant.

In this study, we investigated the genotoxic effects of Ag NPs on TK6 cells post UVC irradiation with qRT- PCR. In the present study, showed that treatment of TK6 cells with Ag NPs can significantly increase *H2AX* gene expression in the absence of UVC irradiation after 1 hour and 24 hours post UVC irradiation (Fig.3A). This trend was observed in all Ag NPs concentrations. Also the results show that UVC irradiation alone can increase *H2AX* gene expression in TK6 cells (Fig.3B). We observed that *H2AX* expression was increased 1 hour and 24 hours after UVC irradiation (P<0.01). The gene expression in TK6 cells after co-treatment with Ag NPs and UVC irradiation was compared with their control groups. *H2AX* gene expression after being treated with 10 and 5 µg/ml of Ag NPs post UV exposure was significantly increased, both 1 hour and 24 hours after UVC treatment (Fig.4A, B, P<0.05). 

## Discussion

In recent years, increasing usage of Ag NPs has led to the need for evaluating their cytotoxicity and genotoxicity. Recently, the cytotoxic effects of Ag NPs in various types of cells such as Hella cells (20), human glioblastoma cells (U251) (21), BRL 3A rat liver cells (22) have been evaluated. The sun’s UV radiation can have many biological effects, including changes in the structure of DNA, proteins and other biological molecules (23, 24). For instance, Glover et al. (25) showed that after irradiation TK6 cells with UVC, the sensitivity to DNA damage was increased. It also increased the amount of apoptosis, delayed DNA repair and caused changes in the expression of P53-target genes. 

Xu et al. (20) showed that the viability of Hella cells treated with Ag NPs (0-30 µg/ml Ag NPs) were decreased after 24 and 48 hours. Similarly, an increase in cytotoxicity was observed in cells treated with Ag NPs by Hussain et al. in BRL 3A rat liver cells (22). In the present study, cytotoxicity of Ag NPs combined treatment with UVC irradiation in TK6 cells revealed that combined treatment can reduce TK6 viability at two separate time points (1 hour and 24 hours) post UVC irradiation. Furthermore, MTT colorimetric assay showed a time dependent reduction of cell viability of TK6 cells. A decrease in survival rate was observed at all NP concentrations, which was in line with previous studies (10, 20, 22, 26). 

There are several studies that have evaluated the effects of UVA and UVB. For example, the genotoxicity of Ag ions and UVB combined treatment was investigated by Zhao et al. (2). They showed that UVB and Ag ions simultaneous exposure in a human keratinocyte cell line, HaCaT, can induce DNA breaks by measuring an increase in *H2AX*. Induction of marked toxic effects against bacteria through combined treatment using Ag NPs and UVA was also investigated by Zhao et al. (27). .-*H2AX* expression was measured 1 hour and 24 hours post ionizing radiation by Li et al. (28) and they observed that expression of *H2AX* significantly increased 1 hour post irradiation. Also Zhang et al. (29) showed that after whole body irradiation of mice, *H2AX* mRNA expression increased significantly in comparison with control groups. Recently .-*H2AX* foci, gene expression, miRNA and protein profile were used as biomarkers for radiation (30-34). *H2AX* gene expression is proportional to the early cellular response to DSBs (18), which can be induced by UV irradiation (35). Therefore, due to the release of silver ions from Ag NPs that leads to *H2AX* gene expression; the present study aimed to investigate the potential genotoxicity of Ag NPs along with UV exposure by measuring *H2AX* gene expression using qRT-PCR. 

Without applying UVC radiation *H2AX* gene expression increased with the increase in nanoparticle concentration. Results show that UVC alone can induce a significant enhancement in *H2AX* gene expression. When the cells were co-exposed to Ag NPs and UVC, a significant increase in relative gene expression in comparison with its control group was observed 1 hour after irradiation with 10 µg/ml Ag NPs. As post treatment time increased from 1 hour to 24 hours, we found that there was a significanct increase in *H2AX* gene expression (in 5 µg/ml Ag NPs) in comparison to its control group. In a study by Glover et al. (25) the effects of DNA damage response in TK6 cells treated with 12-O-Tetradecanoylphorbol-13-acetate (TPA)+UVC was evaluated using *γ-H2AX* formation in various times, after UVC irradiation (0-24 hours). Results showed that cell treatment with TPA and UVC caused a significant increase in *γ-H2AX*, 2 hours after UVC exposure. In our study, we observed *H2AX* synergistic gene expression in 24 hours post UVC treatment in cells treated with 5 µg/ml Ag NPs. 

Uddin et al. (36) evaluated the effect of low concentrations of arsenite and showed that it can increase the risk of skin cancer after UV irradiation. Hence, to investigate the effects of Ag NPs in low concentrations we chose 5 and 10 µg/ml of NPs, and it was observed that in these two concentrations, at 1 hour and 24 hours after UVC irradiation *H2AX* gene expression was increased. Based on these results, co-treatment of TK6 cells with Ag NPs and UVC irradiation can have a synergic effect and significantly increase *H2AX* gene expression. Therefore, the use of Ag NPs and UVC irradiation can be effective in death of cancer cells. This means that Ag NPs can be used as a sensitizing agent for UVC irradiation to combat cancer cells. 

## Conclusion

Try-pan blue and MTT tests revealed that simultaneous use of silver nanoparticles and UVC irradiation can lead to increased cytotoxicity. We have found that exposing human lymphoblastoid TK6 cells to UVC after treatment with increasing concentrations of Ag NPs can induce dose dependent cellular toxicity. In addition, evaluating the in vitro genotoxicity of Ag NPs at different concentrations alongside UVC exposure revealed that UVC irradiation can enhance the genotoxic effects Ag NPs as revealed by increased *H2AX* gene expression. The results of this study show a significant synergistic increase in *H2AX* gene expression could occur in TK6 cells co-exposed to Ag NPs and UVC irradiation. Consequently, combination of Ag NPs and UVC irradiation could be used in cancer therapy. 
